# Binocular Glaucomatous Visual Field Loss and Its Impact on Visual Exploration - A Supermarket Study

**DOI:** 10.1371/journal.pone.0106089

**Published:** 2014-08-27

**Authors:** Katrin Sippel, Enkelejda Kasneci, Kathrin Aehling, Martin Heister, Wolfgang Rosenstiel, Ulrich Schiefer, Elena Papageorgiou

**Affiliations:** 1 Computer Engineering Department, University of Tübingen, Tübingen, Germany; 2 Centre for Ophthalmology, Institute for Ophthalmic Research, University of Tübingen, Tübingen, Germany; 3 Competence Centre “Vision Research”, Study Course “Ophthalmic Optics/Audiology”, University of Applied Sciences Aalen, Aalen, Germany; 4 Department of Ophthalmology, University of Leicester, Leicester Royal Infirmary, Leicester, United Kingdom; Jilin University, China

## Abstract

Advanced glaucomatous visual field loss may critically interfere with quality of life. The purpose of this study was to (i) assess the impact of binocular glaucomatous visual field loss on a supermarket search task as an example of everyday living activities, (ii) to identify factors influencing the performance, and (iii) to investigate the related compensatory mechanisms. Ten patients with binocular glaucoma (GP), and ten healthy-sighted control subjects (GC) were asked to collect twenty different products chosen randomly in two supermarket racks as quickly as possible. The task performance was rated as “passed” or “failed” with regard to the time per correctly collected item. Based on the performance of control subjects, the threshold value for failing the task was defined as *μ+3*σ (in seconds per correctly collected item). Eye movements were recorded by means of a mobile eye tracker. Eight out of ten patients with glaucoma and all control subjects passed the task. Patients who failed the task needed significantly longer time (111.47 s ±12.12 s) to complete the task than patients who passed (64.45 s ±13.36 s, t-test, p<0.001). Furthermore, patients who passed the task showed a significantly higher number of glances towards the visual field defect (VFD) area than patients who failed (t-test, p<0.05). According to these results, glaucoma patients with defects in the binocular visual field display on average longer search times in a naturalistic supermarket task. However, a considerable number of patients, who compensate by frequent glancing towards the VFD, showed successful task performance. Therefore, systematic exploration of the VFD area seems to be a “time-effective” compensatory mechanism during the present supermarket task.

## Introduction

Glaucoma is a progressive optic neuropathy leading to characteristic visual field defects, and blindness if left untreated. Glaucoma usually leads to (arcuate) visual field defects that follow the course of the affected retinal nerve fibers. In advanced stages of glaucoma the areas of monocular field defects may spatially coincide and thus result in binocular field loss. The central visual field (VF) and the visual acuity are usually spared even up to end-stage glaucoma.

Patients with binocular glaucomatous visual field loss may experience severe difficulties in activities of daily living such as reading, mobility, or driving [1]–[8]. Furthermore, due to demographic aging, the number of people suffering from glaucoma is expected to increase. According to Quigley and Broman 2006 [9] the number of people who will be bilaterally blind from open-angle glaucoma is expected to rise to 5.9 million by 2020. Therefore, the impact of glaucoma on everyday activities and on the quality of life of affected individuals is being investigated intensively during the last years. Many studies have assessed the impairment of patients with glaucoma in everyday activities by means of questionnaires [5], [6], [10]–[17], simulators, or under laboratory conditions [18]. Their results suggest that visual search behavior of affected subjects plays a decisive role in coping with everyday activities. The most realistic attempt to assess the functional impairment of patients with glaucoma in everyday activities is by conducting real-world experiments, mostly regarding driving fitness [1], [3], [19], [20]–[22]. The above previous studies agree on certain aspects: (i) the task performance varies among individuals, (2) binocular visual field defects do not always lead to poorer performance, and (3) binocular visual field defects can be compensated by effective head and eye movement strategies. However, the results of driving studies may not reflect patients’ visual behavior in other everyday activities, because compensatory gaze patterns are highly specific and intrinsically related to the specific task [23]. Furthermore, there is variability in patients’ compensatory strategies during activities of daily living. Since real-world experiments are expensive, time-consuming, and difficult to standardize, to date only few everyday activities have been investigated.

A common scenario in everyday life involving visual search is shopping, where we permanently search for specific items. Most prior work on visual search during shopping has aimed at understanding the consumer’s psychology, e.g., [24]–[27]. To the best of our knowledge no studies investigating the visual search of patients with binocular glaucomatous visual field defects during shopping tasks have been conducted up to date. Assessment of activities of daily living is necessary for a better understanding of the compensatory strategies of patients with binocular glaucoma. Hence, evaluation of visual exploration during daily activities will be helpful in evaluating global, vision-targeted QOL, in order to improve the correlation between visual function and its perception, design training strategies for improvement of daily functioning, and develop examination tools for usage in the clinical setting.

Therefore, the aim of this pilot study was to assess the visual search performance of patients with binocular glaucomatous visual field loss in a supermarket special offer search task. The patients’ performance was compared to that of healthy-sighted control subjects. Furthermore, we investigated the factors affecting task performance and studied features of the visual search strategy. We hypothesize that the performance of patients with binocular glaucoma is not primarily associated to the extent of the visual field defect, but is mainly related to their visual scanning strategy.

## Methods

### Participants

Twenty participants were enrolled in this study: ten patients with glaucoma (we refer to this group as GP, age 60.7±8.7 years) and ten healthy-sighted control subjects (GC, age 59.9±9.1 years), matched with respect to age and gender (Table S1). All participants were recruited by the Neuro-Ophthalmology service of the University of Tübingen.

To be included in the study all participants were required to be at least 18 years old, have a Minimental Status Examination Score above 24, to have the ability to understand and comply with the requirements of the study, and normal function and morphology of the anterior visual pathways, as evaluated by ophthalmological tests (fundus and slit-lamp examinations, ocular alignment, ocular motility). Color vision should be normal using the Ishihara isochromatic color plates. The age- and gender-matched control subjects should additionally have normal visual fields, normal cup-to-disc ratio (less or equal to 0.5) and no history of brain injury or physical impairment. The best corrected monocular distant visual acuity of control subjects should be >20/20 for those aged-up to 60 years, >20/25 for those aged between 60–70 years and >20/33 for those aged more than 70 years. Patients’ best corrected monocular distant visual acuity should be at least 20/40. Glaucoma patients had a confirmed diagnosis of primary open angle glaucoma based on optic nerve damage and visual field loss. Only glaucoma patients with advanced binocular visual field loss were included (stages II-IV according to the Aulhorn classification [28]). Mean time since first glaucoma diagnosis was 12.3 years (±7.47 years).

Visual fields were assessed by means of binocular semi-automated 90^o^ kinetic perimetry (SKP) obtained with the OCTOPUS 101 Perimeter (background luminance 10 cd/m^2^, angular velocity 3°/s, Fa. HAAG-STREIT, Koeniz, Switzerland). We used the binocular kinetic visual field because it provides more realistic information about the visual field which is needed in daily activities [29].

The research study was approved by the Institutional Review Board of the University of Tübingen (Germany) and was performed according to the Declaration of Helsinki. Following verbal and written explanation of the experimental protocol, all subjects gave their written consent with the option of withdrawing from the study at any time. Clinical Trial Registration http://www.clinicaltrials.gov. Unique identifiers: NCT01372319, NCT01372332.

### Supermarket Search Task

**Figure 1:**
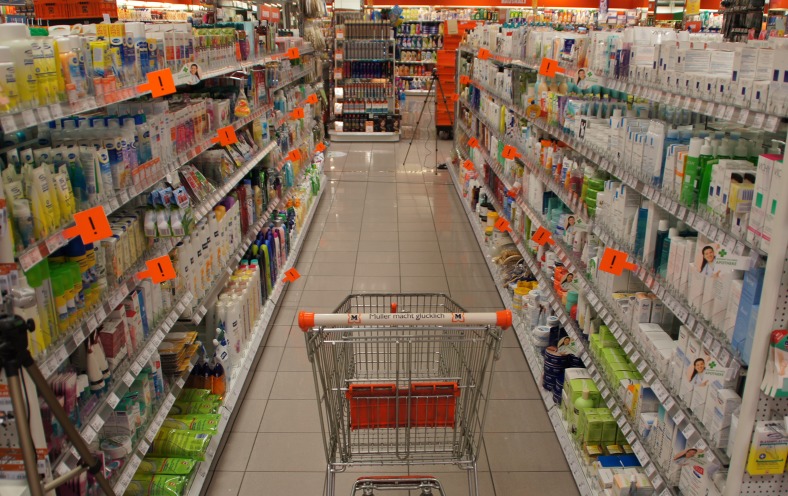
The drug store corridor with all marked special-offer products (orange signs) on two racks (each containing six shelves). Two cameras (marked by blue circles) at the beginning and the end of the corridor were used to record navigation of the subjects during the task.

The experiment was performed in a drugstore in the city center of Tübingen. Twenty different special-offer products were chosen randomly among other products in two racks (one right-hand and one left-hand rack) along a corridor of 7.5 m length and 1.3 m width (Figure 1). Furthermore, each of these racks included 6 shelves at different heights as presented in Figure 1. The products of interest varied in color, shape, and size and were marked by orange tags (Figure 1). The products were distributed homogeneously over height and width of both shelves.

The subjects were asked to collect all marked products in both shelves as quickly as possible by walking through the corridor only once. Hence, the main task was to locate the orange tags and collect the above standing product. Eye movements were recorded by means of a Dikablis mobile eye tracker (Ergoneers Inc., Manching, Germany). The eye-tracking device is a light-weighted, head-mounted monocular unit, which does not interfere with glasses.

The supermarket search task was repeated four times by each participant. In order to record the eye movements of subjects during the search task, an eye tracker was worn during the first run. The remaining three runs were performed without eye-tracking equipment due to a very tight time schedule. More specifically, the experiments were conducted in the mornings between 7 and 9 a.m. (i.e., before the supermarket opened). During the last three runs of a subject, the eye-tracking device was calibrated for the next subject. For each trial, the time, the item description, the number of collected items, and the number of wrongly collected items were documented.

### Performance Assessment

The performance of subjects was assessed by means of the following parameters: (i) average number of correctly collected items *N_c_* over all runs, (ii) average performance time *t* over all runs, and (iii) average time per correctly collected item *T_C_ = t/N_C_*.

#### Passing criterion

The *T_C_* values followed a normal distribution with (mean) *μ* = 3.28 s and (SD) σ = 0.88 s in the control group (Shapiro-Wilk test). Based on performance of the control subjects, the threshold value of *T_C_* for failing the task was chosen as *T_C_*-failed = *μ+3*σ. More specifically, *T_C_*-failed = 5.92 s, i.e., a subject who needed longer than 5.92 seconds per correctly collected item failed the task.

In order to identify parameters associated with successful task performance, *N_C,_ t* and *T_C_* were compared across glaucoma control subjects who passed (GCp), glaucoma patients who passed (GPp), and glaucoma patients who failed (GPf) the task by one-way ANOVA. Subsequent post-hoc comparisons were performed using the Tukey’s HSD test. As multiple tests were carried out, the significance level was adjusted using a Bonferroni correction to an alpha-level of 0.05 for multiple comparisons. All data sets were tested for normality by the Shapiro-Wilk test; for non-normally distributed data, the Mann-Whitney U test and the Kruskal-Wallis test for multiple comparisons were used. In addition, dependencies between eye-movement-related parameters and performance-related parameters (N_c_, t, and T_c_) were also assessed using linear regression (Pearson Correlation Coefficient) in the patient group. Matlab R2013b was used for data analysis.

In order to investigate the effect of the visual field defect on task performance, the size of the binocular visual field defect was calculated from measurements obtained by means of binocular semi-automated 90^o^ kinetic perimetry (SKP) as described above (see Table S1). Only the stimulus III/4e was used, since this is a functionally relevant target that is typically used to define driving fitness and legal blindness in Germany.

### Analysis of Eye Movement Data

Eye movements were recorded using the D-Lab software tool (Ergoneers Inc, Manching, Germany) at a frequency of 25 frames per second. The recorded data was analyzed using both D-Lab and self-developed algorithms. For the detection of fixation clusters and saccades from raw eye-tracking data we applied a Bayesian learning algorithm [30]–[32]. In order to quantify the frequency and duration of saccades towards the area of visual field defect or towards the peripheral visual field, we superimposed the area of visual field defect as Area Of Interest (AOI) for each participant. These models were transferred into D-Lab to analyze the viewing behavior of participants towards such regions in terms of glance proportion and frequency. We assessed the following gaze-related parameters:

#### Horizontal Gaze Activity (HGA)

In order to investigate the horizontal exploration ability of a subject, we assessed the horizontal standard deviation of the pupil, which was expressed as Horizontal Gaze Activity (HGA).

#### Glance Proportion in percentage (PGP)

PGP describes the percentage of glance duration in a defined AOI during a given time interval. We computed the PGP for the area of visual field defect (PGP-VFD), for the visual field area beyond 30° (PGP-30c), and for the visual field area beyond 60° (PGP-60c).

Glance Frequency (GF). GF describes the average number of glances towards a defined AOI during the time unit of one second. Similar to PGP, we computed the GF for the area of visual field defect (GF-VFD), the visual field area beyond 30° (GF-30c), and the visual field area beyond 60° (GF-60c).

## Results

### Task performance

**Figure 2:**
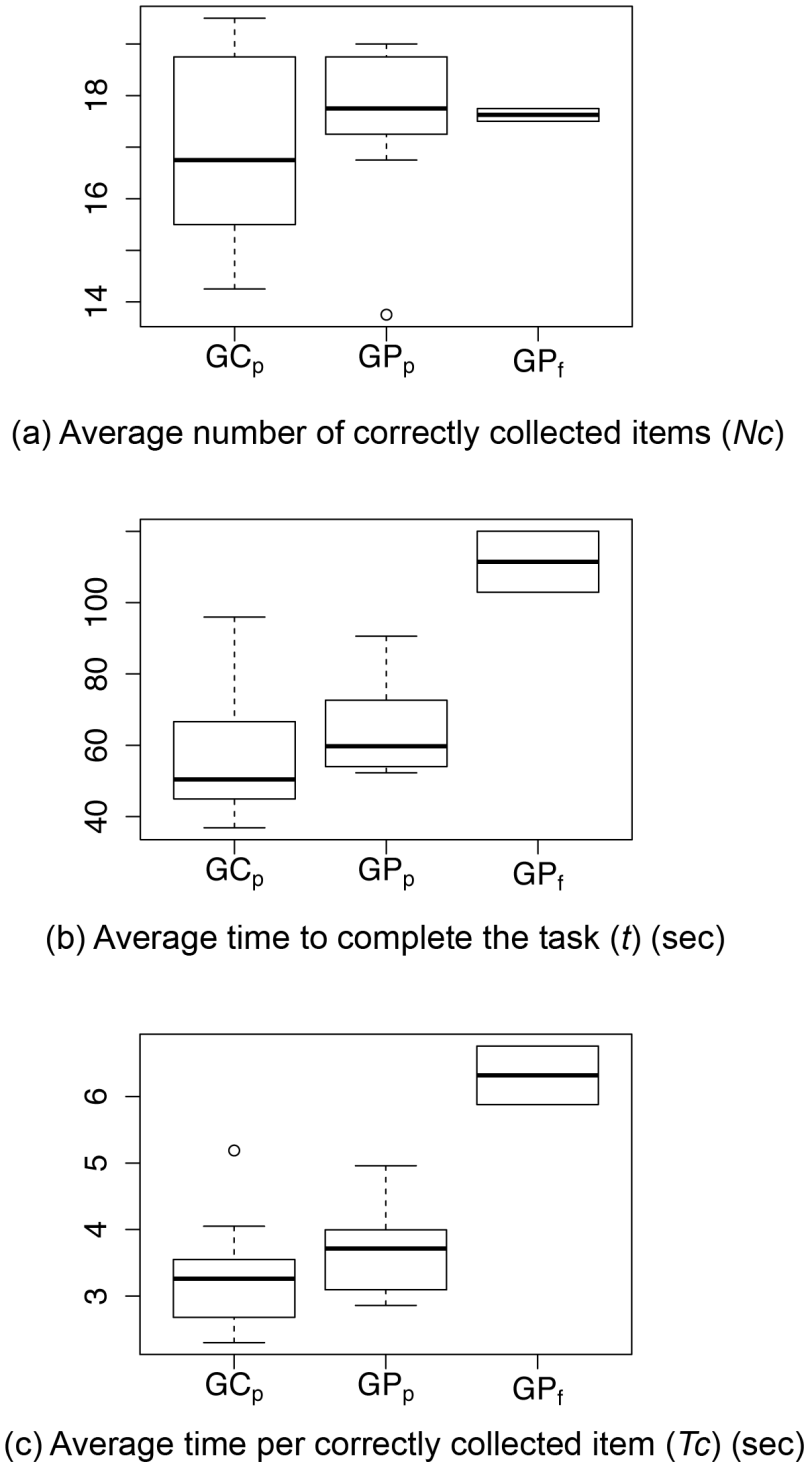
Value range for (a) the average number of correctly collected items over all runs, (b) the average time needed to complete the supermarket search task over all runs, and (c) the average time (over all runs) per correctly collected item. The participant subgroups are marked by GCp/GPp (glaucoma controls/patients who passed), GPf (glaucoma patients who failed).

For each subject subgroup, Figure 2 presents (a) the average number of correctly collected items *N_C_*, (b) the average time *t* to complete the task, and (c) the average time per correctly collected item *T_C_*.

#### Number of correctly collected items (N_C_)

None of the subjects was able to collect all 20 items successfully (Figure 2a). However, contrary to our expectation, the general task performance regarding Nc was good, e.g., the worst performer collected 13 out of 20 items. Furthermore, we found no significant differences in the number of correctly collected items between the glaucoma patients and control subjects. For GPp, we found a strong negative correlation between N_c_ and the size of the visual field defect (VFDsize) (r = 0.81), indicating that the number of correctly collected objects decreases with increasing size of the visual field defect.

The number of wrongly collected items was very small, there were overall only 7 wrongly collected items for all participants and all runs (three times one error, two times two errors), therefore no further analyses were performed.

#### Average performance time (t)

Regarding the overall performance time, we found a highly significant difference between the glaucoma subgroups GCp, GPp, and GPf (p<0.001, Table 1). GCp needed on average 55.94 s to complete the task, GPp 64.45 s, while GPf needed almost twice as long as GPp to complete the task. In summary, control subjects (GCp) and patients who passed the supermarket search task (GPp) needed considerably less time to complete the trial than patients who failed the task (GPf).

**Table 1:** Performance and gaze-related parameters for glaucoma control subjects who passed (GCp), glaucoma patients who passed (GPp), and glaucoma patients who failed the task (GPf).

	GC_p_ - GP_p_ - GP_f_
**N_c_**	n. s.
**T**	***
**T_c_**	***
**HGA**	n. s.
**PGP - 60^c^**	n. s.
**PGP - 30^c^**	n. s.
**PGP – VFD**	n. s.
**GF - 60^c^**	n. s.
**GF - 30^c^**	n. s.
**GF – VFD**	*

Statistical comparisons were made between the groups.

*p<0.05; ***p<0.001, n.s: indicates non-significant comparisons.

#### Average time per correctly collected object (T_C_)

According to the passing threshold of *T_C_* = 5.92 sec (see Section Methods) all control subjects completed the supermarket search task successfully and displayed significantly shorter t and Tc values than the patient group. The success rate for glaucoma patients was 80%. Since the parameter *T_c_* depends on *t*, we found the same significant differences between subject subgroups as for *t.* More specifically, subjects who passed the task (GCp) needed significantly shorter time per collected item than subjects who failed the task (p<0.001, Table 1).

In summary, these results suggest that binocular glaucomatous visual field loss is mainly associated with longer search time. However, a subgroup of patients performed indistinguishably from conytol subjects, possibly by means of efficient gaze compensation.

### Gaze-related parameters

#### Horizontal Gaze Activity (HGA)

Contrary to our expectations, no difference was found in the horizontal gaze activity between the participant subgroups (see Table 1).

Furthermore, no significant relationship was found between HGA and Nc or the average time per correctly collected object (Tc) in any of the subject subgroups. Thus, it seems that HGA does not influence task performance.

#### Proportion of Glances in Percent (PGP)

There was no significant difference between the subject subgroups regarding the proportion of glances beyond the 30° visual field (PGP-30c) and towards the visual field defect (PGP-VFD), see Table 1.

Furthermore, we found a strong positive relationship between Nc and the PGP towards the VFD in GPp (r = 0.77), which indicates that patients who passed the task demonstrated more efficient exploration of the VFD area, i.e., the longer duration of glances towards the VFD enables the detection of more target objects.

#### Glance Frequency (GF)

There was no significant difference between the subject subgroups in glance frequency (GF) beyond 30° (GF-30c) and beyond 60° (GF-60c).

A significant difference in GF towards the visual field defect area (GF-VFD) was found between GPp and GPf (Table 1). Glaucoma patients who passed (GPp) performed more glances towards their visual defect area than patients who failed (GPf), (p<0.05, Table 1).

## Discussion

We investigated the performance of patients with binocular glaucomatous visual field loss in comparison with healthy-sighted control subjects during a special-offer supermarket search task. Our study is novel, because visual search behavior of patients with binocular visual field loss during real-life tasks has not been quantified by means of eye-tracking equipment so far. With a pass rate of 80%, a considerable number of patients with glaucoma managed to pass the test despite their binocular visual field loss. This finding is in accordance with prior studies [3], [19] reporting the performance of patients with glaucomatous visual field defects in driving tasks.

With regard to the number of correctly collected items, no significant differences were found between the subject subgroups. Thus, when time is not restricted, patients with glaucomatous visual field loss perform indistinguishably from a control group. However, in many real-life scenarios there is time pressure. Therefore, we defined a time-related passing criterion based on the performance of normal subjects. According to this criterion, the control group needed on average a shorter time per correctly collected item (*Tc*) than the patient group. Although binocular glaucomatous visual field defects is in general associated with longer search time, a subgroup of patients completed the task within the defined time period (GPp), while patients who failed (GPf) needed considerably longer time to complete the task. On the other hand, glaucoma patients who passed the test (GPp) displayed more efficient exploration of the visual field defect area. Therefore, they managed to locate the target object faster than glaucoma subjects who failed. One might expect that increased scanning activity could be time-demanding and lead to longer task duration. However, our findings suggest that systematic visual search towards the areas, which are considered to be “problematic” in glaucoma patients, namely the areas of the VFD, can be time-effective. In contrast, failure to systematically scan those areas leads to disorganised scan patterns that prove to be time-consuming and ineffective in everyday tasks.

Furthermore, glaucoma patients who passed the task (GPp) showed a gaze bias towards their visual defect area, as indicated by the higher glance frequency (GF) towards the VFD. By directing their gaze towards the area of visual field loss, patients manage to bring more visual elements into their seeing field and thus detect more target objects, which might be obstructed by the VFD. This result is in accordance with our recent on-road study, where we also found increased glances towards the VFD area in patients with binocular visual field loss who passed a driving test [19].

There is limited literature on gaze patterns of patients with glaucomatous visual field loss during real-life tasks. In accordance with our results, Crabb et al. [33] have also reported that patients with bilateral glaucomatous visual field loss made more saccades than a control group when viewing driving scenes during a hazard perception test, in an attempt to compensate for their restricted field of view [33]. These authors also suggested that a glaucomatous visual field defect may cause detection deficits, which could be compensated by gaze movements, since there were revealing cases where patients failed to see a hazard in relation to their binocular visual field defect. Similar detection deficits and longer reaction times in patients with mild to severe glaucoma were also reported in the driving simulator study conducted by Vega et al. [34] in 2013. These authors did not find compensatory visual search patterns for patients [34]. However, gaze compensation was possibly less required in the above study, because the driving simulation did not include other traffic [34]. Despite this, only nine out of 23 participants had binocular field loss.

Vargas-Martin and Peli [35] reported in 2006 that patients with severe VFD due to retinitis pigmentosa (RP) exhibited narrower horizontal eye-position dispersions than normal subjects during walking in real environments, due to the absence of peripheral visual stimulation and the simultaneous use of head movements [35]. In our study, the horizontal extent of exploratory eye movements did not differ between the participant subgroups, as indicated by similar HGA values, which was calculated from the horizontal standard deviation of the pupil. A possible explanation for differences found in [35] is that the above study included RP patients with more severe visual field loss (less than 20^o^ total extent of horizontal and vertical visual field in both eyes). In addition, visual field defects in RP are due to damage of the photoreceptors and the retinal pigment epithelium, while glaucoma is associated with a lesion of the retinal ganglion cells. Hence, the site of lesion in the visual pathway and the different adaptation state (nyctalopia in RP) may lead to distinct exploratory patterns. Furthermore, the above study included a walking route with segments in unfamiliar indoor environments and city streets [35]. In contrast, our participants had to detect and collect specific items, which were placed in expected spatial locations, namely the two supermarket racks. Therefore they could focus on the specific task and their scanning strategy was therefore probably more organized and target-oriented due to possible top-down influences. Compensation was achieved by means of more frequent and longer glances towards the VFD and towards the peripheral visual field. This finding supports the hypothesis presented by Luo et al. [36], who indeed stressed the importance of the top-down mechanism influence on eye-movement control. They also found very frequent beyond-VF saccades in people with tunnel vision, which could not be triggered by instantaneous visual salience [36]. In addition, the nature of the present everyday task would indeed point towards implementation of top-down information based on prior knowledge and intention, in order to provide guidance to eye movements [37]. We therefore agree with the study of Luo et al. [36] that scanning of non-seeing areas may not necessarily lead to instant, accurate detection of the target. However, this approach increases the chances of bringing the desired targets into the seeing field, in order to guide saccades based on bottom-up saliency.

On the other hand, Wiecek et al. 2012 [38] found that patients with peripheral visual field loss (PVFL) due to glaucoma or optic nerve drusen showed a biased directional distribution that was not directly related to the locus of vision loss, challenging feed-forward models of eye movement control. In addition, total search duration, fixation duration, saccade size, and number of saccades showed no difference between PVFL patients and normal subjects. This inconsistency with our results may be attributed to the difference in stimuli (e.g., 26°×11° images versus natural environment), design of the experiment (use of a chin rest versus free navigation) and monocular versus binocular visual search [38]. Finally, the authors have explained that their visual search task was specifically designed to minimize the role of top-down factors and observers frequently made eye movements into areas of vision loss, although this finding did not reach statistical significance [38].

Task duration in the current experiment was longer in glaucoma patients than in controls, which confirms prior work by Smith et al. [18], suggesting slower performance for glaucoma patients compared to control subjects during visual search tasks. Prior studies by Cornelissen et al. (2005) [39] and Coeckelbergh et al. 2002 [40] on the impact of central and peripheral visual field defects in visual search tasks also reported an increase in search times in patients compared to controls and an additional increase in the number of errors with increasing visual field deficits. Our results are in accordance with these findings, since we also found that the number of correctly collected objects (Nc) decreases with an increase in the glaucomatous visual field defect size.

In summary, gaze pattern analysis revealed that successful task performance of glaucoma patients is associated with longer and frequent glancing towards the VFD.

### Limitations of the study

The above findings should be interpreted in the light of some study limitations. Despite the considerable total number of participants (20 subjects), the number of participants in the subgroups was relatively small. Thus, further studies involving a larger number of subjects have to be conducted. An important issue that will be investigated in our future work concerns the role of head movements during natural visual search tasks. From driving studies there is evidence that patients with binocular VFD compensate by head movements, especially when the task requires exploration of a wide horizontal FOV [19]. In the present task, no differences were found in HGA, which expresses the horizontal standard deviation of the pupil and is an indirect measure of eye movement (saccadic) amplitudes. This points towards the use of head movements in order to reach eccentric locations of the field of view. Therefore, in future studies we will integrate head tracking devices to study the contribution of head movements in natural search tasks. In addition, one should address the motor component of the present task, when trying to interpret trial duration. Subjects were required to identify the item location, then collect the item, and finally move towards the end of the corridor. Although there is no reason to assume any motor differences between groups, the trial duration included the visual search plus the motor response. Finally, some of the participants had some degree of macular sparing, which might affect gaze movement strategies. However, due to the free navigation of participants in a natural environment and the need to locate targets in the far periphery, immediate visual input from the area of macular sparing is unlikely, as also shown by the gaze bias towards the area of the VFD.

## Conclusion

Binocular glaucomatous visual field loss may critically interfere with quality of life. In a special-offer supermarket search task we investigated the performance of ten patients with glaucoma and ten healthy-sighted, age- and gender-related control subjects. 80% of the patients completed the task successfully despite their visual impairment. We found that binocular glaucomatous visual field loss was on average associated with longer search time. However, analysis of eye-tracking data revealed that patients who are able to compensate for their visual field defect employ frequent glancing towards the area of the visual field defect.

## Supporting Information

Table S1 **Demographic data and visual fields of glaucoma patients who participated in the study.** t represents the time since first diagnosis of glaucoma.
(DOCX)
