# Supplementary material for: Binocular Glaucomatous Visual Field Loss and Its Impact on Visual Exploration - A Supermarket Study
Source: PLoS One. 2014 Aug 27;9(8):e106089. doi: 10.1371/journal.pone.0106089 (PMC4146567; doi:10.1371/journal.pone.0106089)
Supplement: Table S1 — Demographic data and visual fields of glaucoma patients who participated in the study. t represents the time since first diagnosis of glaucoma. (DOCX) [file pone.0106089.s001.docx]

# APPENDIX

| **ID** | **gender** | **age** | **t (yrs)** | **pathogenesis** | **VFD** | **VFD size** |
| --- | --- | --- | --- | --- | --- | --- |
| 61 | m | 74 | 5 | normal pressure glaucoma | 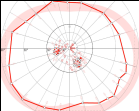 | 5,26% |
| 63 | m | 59 | 15 | open angle glaucoma | 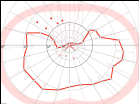 | 42,11% |
| 65 | m | 45 | 11 | open angle glaucoma | 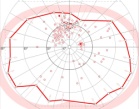 | 6,58% |
| 67 | f | 71 | 5 | open angle glaucoma | 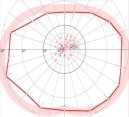 | 2,63% |
| 69 | f | 62 | 5 | glaucoma | 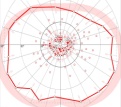 | 11,84% |
| 71 | m | 66 | 18 | open angle glausoma | 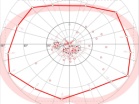 | 5,26% |
| 73 | m | 55 | 23 | open angle glaucoma | 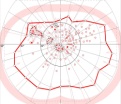 | 13,16% |
| 75 | f | 59 | 14 | normal pressure glaucoma | 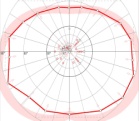 | 2,63% |
| 77 | m | 52 | 23 | open angle glaucoma | 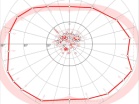 | 10,53% |
| 79 | f | 64 | 4 | open angle glaucoma | 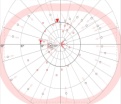 | 19,74% |

Table S1: Demographic data and visual fields of glaucoma patients who participated in the study. t is the time since first diagnosis of glaucoma.
